# Expression of RARRES1 and AGBL2 and progression of conventional renal cell carcinoma

**DOI:** 10.1038/s41416-020-0798-6

**Published:** 2020-04-20

**Authors:** Lehel Peterfi, Daniel Banyai, Maria V. Yusenko, Thea Bjercke, Gyula Kovacs

**Affiliations:** 1 Department of Urology, Medical School, University of Pecs, Pecs, Hungary; 2 Institute of Biochemistry, University of Muenster, Muenster, Germany; 3 Medical Faculty, Ruprecht-Karls-University, Heidelberg, Germany

**Keywords:** Prognostic markers, Renal cell carcinoma

## Abstract

**Background:**

Approximately 15% of clinically localised conventional renal cell carcinoma (RCC) will develop metastasis within 5 years of follow-up. The aim of this study was to identify biomarkers predicting the postoperative tumour relapse.

**Methods:**

Tissue microarrays of conventional RCC from a cohort of 691 patients without metastasis at the time of operation were analysed by immunohistochemistry for the expression of carboxypeptase inhibitor RARRES1 and its substrate carboxypeptidase AGBL2. Univariate and multivariate Cox regression models were addressed to postoperative tumour relapse and the metastasis-free survival time was estimated by Kaplan–Meier analysis.

**Results:**

In multivariate analysis, the lack of staining or cytoplasmic staining of RARRES1 was a significant risk factor indicating five times higher risk of cancer relapse. Combining its co-expression with AGBL2, we found that RARRES1 cytoplasmic/negative and AGBL2-positive/negative staining is a significant risk factor for tumour progression indicating 11–15 times higher risk of cancer relapse, whereas the membranous RARRES1 expression, especially its co-expression with AGBL2, associated with excellent disease outcome.

**Conclusions:**

RARRES1 and AGBL2 expression defines groups of patients at low and high risk of tumour progression and may direct an active surveillance to detect metastasis as early as possible and to apply adjuvant therapy.

## Background

Renal cell carcinoma (RCC), the most frequent kidney cancer, has a high mortality, and approximately 40–50% of the patients have a metastasis at the time of first observation or will develop metastatic disease during the postoperative course.^1,2^ Metastatic conventional RCC is resistant to radiation and cytotoxic therapy. Recent drug therapies targeting vascular endothelial growth factor and mammalian target of rapamycin pathways or applying biologic and immunomodulatory molecules including small molecule kinase inhibitors and specific antibodies can only prolong the life of patients with metastatic disease.^3^ In recent years, as a result of widespread use of imaging techniques, a growing number of patients are diagnosed with incidentally detected small renal mass confined to the kidney, the overwhelming majority of which are conventional RCC.^4,5^ However, approximately 15% of clinically localised conventional RCCs operated with curative intent will develop metastasis within 5 years of follow-up.

By applying global gene expression analysis using the Affymetrix array, we have identified increased expression of several genes including the retinoic acid receptor responder 1 (RARRES1) in rapidly progressing conventional RCC. RARRES1 is a transmembrane carboxypeptidase inhibitor that interacts with the carboxypeptidase ATP/GTP-binding protein like 2 (AGBL2).^6^ Decrease or lack of RARRES1 expression, frequently caused by promoter hypermethylation, has been described in prostate, endometrial, head and neck, nasopharyngeal, colorectal and gastric cancer cell lines suggesting that RARRES1 is a tumour-suppressor gene.^7–13^ Studies carried out on human tumour tissues yielded controversial results. A strong downregulation of RARRES1 have been found in colorectal carcinoma comparing to benign adenoma and normal colon tissue.^14^ On the other hand, high expression of RARRES1 in inflammatory breast cancer tissue correlated with shorter survival of patients.^15^ An immunohistochemical study on distinct types of renal cell tumours suggested a prognostic value of nuclear and cytoplasmic RARRES1 expression.^16^ The relevance of RARRES1 and AGBL2 expression in conventional RCC is not yet known.

The aim of this study was to evaluate the expression of RARRES1 and AGBL2 and progression of conventional RCC in a cohort of patients without clinically detectable metastasis at the time of operation.

## Methods

### Study design and data collection

We have enrolled 691 patients in this study, who underwent radical or partial nephrectomy for conventional RCC between 2000 and 2013 at the Department of Urology, University of Pecs, Hungary. None of the patients had a detectable metastatic disease at the time of operation. Data on regular follow-up and tumour relapse were obtained from Tumour Registry of the Department of Urology. Follow-up was defined as a time from the operation until the last recorded control or cancer-specific death. Patients who died from causes other than RCC are not counted in this measurement. Preoperative clinical staging included abdominal and chest computed tomography (CT) scans. Bone scans and brain CT scans were carried out only when indicated by clinical signs. The presence of nodal metastasis was confirmed by histological, whereas distant metastases by radiographic examination. In the postoperative period, patients were observed every 6 months by abdominal ultrasound and measurement of serum creatinine and estimated glomerular filtration rate and yearly by CT. The histological diagnosis and tumour, node, metastasis (TNM) classification were re-evaluated by a genitourinary pathologist (GK) according to the Heidelberg and TNM classification systems by applying a 1–3 tumour grading system and taking into account the tumour necrosis as well.^17,18^ We restrained to the Heidelberg Classification because it is based on robust tumour-specific genetic alterations. According to this classification, approximately 70–80% of conventional RCCs are composed of “clear” cells and the rest as “eosinophilic” (earlier “granular”) cells.^19^ The collection and use of all tissue samples for this study was approved by the Ethics Committee of the University Pecs, Hungary (No. 5343/2014)

### Affymetrix array analysis

For the global gene expression analysis, we selected 12 conventional RCC leading to death of patients within 3 years of follow-up and another 12 tumours from patients without detectable tumour progression within 8 years of follow-up. Moreover, we included papillary and chromophobe RCC, renal oncocytoma and childhood tumours in the panel. Gene expression analysis using the Affymetrix Human Genome U133 Plus 2.0 array was carried out as described earlier.^20^ Data of the expression profile have been deposited in NCBI Gene Expression Omnibus under the accession number GSEA (gene set enrichment analysis) 11151.

### Tissue microarray (TMA) and immunohistochemistry

Haematoxylin & eosin-stained slides of conventional RCCs were searched for representative tumour areas and marked for TMA construction. From each tumour, two to five biopsies were taken corresponding to areas of different morphology and/or nuclear grade. TMA was constructed by one of the authors (G.K.) by using a Manual Tissue Arrayer (MTA1, Beecher Instruments, Inc., Sun Prairie, USA) and 0.6-mm core biopsies.

Foetal and adult kidneys and TMAs containing conventional RCCs were used for immunohistochemistry. After dewaxing and rehydration, the 4-µm-thick sections were subjected to heat-induced epitope retrieval in citrate buffer, pH 6.0 in 2100-Retriever (Pick-Cell Laboratories, Amsterdam, The Netherlands). Endogenous peroxidase activity and unspecific binding sites were blocked with 3% hydrogen peroxide containing 1% normal horse serum for 10 min at room temperature. The slides were incubated overnight at 4 °C in moist chamber with the anti-RARRES1 antibody (HPA003892, Sigma Aldrich, Budapest, Hungary) at the dilution of 1:250 and anti-AGBL2 antibody (PA5-39124, ThermoFisher, Budapest, Hungary) at the dilution of 1:100. Horseradish peroxidase-conjugated anti-rabbit secondary antibody (HISTOLSMR, Histopathology Kft, Pecs, Hungary) was applied for 30 min, and colour was developed using the AEC substrate (DAKO, Glostrup, Denmark). Tissue sections were counterstained with Mayer’s haematoxylin (DAKO). For negative control, the primary antibody was omitted. Normal foetal and adult kidney samples included in each TMA were used as positive control. The immunostaining has been carried out twice on two series of slides obtained from the same TMA blocks. The result for RARRES1 was scored as membranous, cytoplasmic or negative staining, whereas for AGBL2 as positive or negative. As >90% of tumour cells were positive in each core biopsy evaluated as positive case, no counting of positive cells was necessary. Slides were evaluated twice by two of the authors (L.P. and G.K.).

### Statistical analysis

Correlations between categorical variables and RARRES1 and AGBL2 expression were estimated by Pearson Chi-square and Fisher’s exact test. Estimates of the cumulative survival distributions were calculated by the Kaplan–Meier method, and the differences between the groups were compared using log-rank test. The significance of clinical–pathological variables was evaluated using the univariate and multivariate Cox proportional hazard regression model. Analysis was performed using IBM SPSS Statistics v.25 for Windows (IBM Inc. Chicago IL, USA). *p* Value <0.05 was considered the limit of statistical significance.

## Results

### Study population

To estimate the prognostic value of RARRES1 expression, we have analysed a cohort of 691 patients having conventional RCCs without clinically detectable metastasis at the first observation. Of the 691 patients, 406 (59%) were males and 285 (41%) females, and the mean age of the cohort was 61.3 ± 11.2 years (range 23–88 years). The average tumour size was 50.2 ± 25.8 mm. During the median follow-up of 73 ± 28 months, tumour relapse was observed in 112 patients (16%). Of the 691 tumours, 511 (74%) were classified as pT1 including 308 (45%) pT1a tumour. The overwhelming majority of RCCs (456 of 691) displayed G1 tumour grade. Regarding the tumour stage, 671 (97%) of the tumours were designated to stages I and II. The pertinent clinical and pathological data are presented in Table 1.

**Table 1:** Association of RARRES1 expression with clinical and pathological parameters (*n* = 691).

	No. of cases (691)	RARRES1 expression			*p* Value
		neg (106)	memb (454)	cyt (131)	
Gender					<0.001
Male	406	74	243	89	
Female	285	32	211	41	
Status					<0.001
AWD	579	78	435	66	
PTR	112	28	19	65	
Size, cm					<0.001
<4	272	30	227	15	
4–7	269	50	163	56	
>7	150	26	64	60	
T					<0.001
pT1	511	71	386	54	
pT2	94	17	53	24	
pT3	86	18	15	53	
Grade					<0.001
G1	456	69	362	25	
G2	180	26	85	69	
G3	55	11	7	37	
Necrosis					<0.001
No	608	93	431	84	
Yes	83	13	23	47	
Stage					<0.001
I	504	71	381	52	
II	167	32	65	70	
III	20	3	8	9	

*PTR* postoperative tumour relapse.

### RARRES1 RNA is upregulated in conventional RCC with progression

**Fig. 1:**
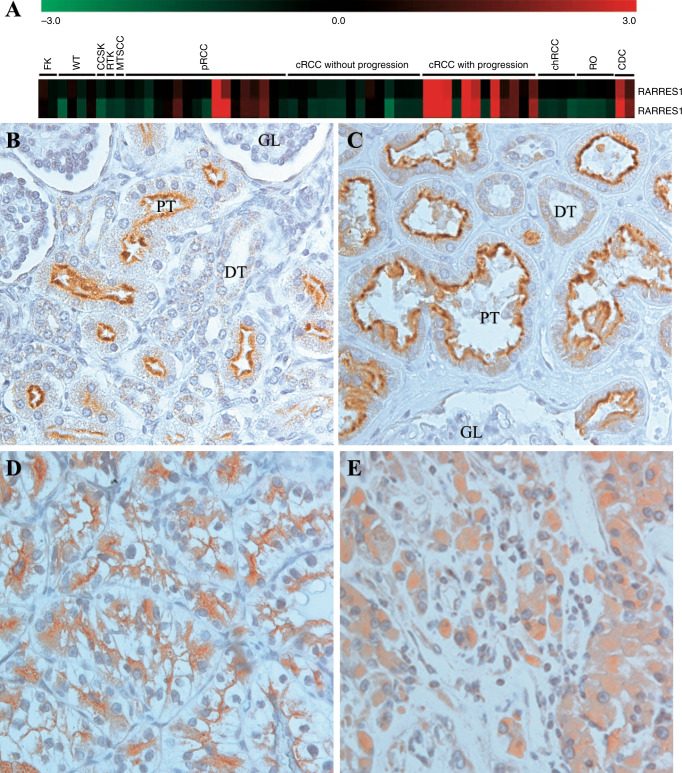
Expression of RARRES1 in conventional RCC. **a** Part of the heat map showing the expression of *RARRES1* in conventional RCC with and without progression and in other types of renal tumours. The increased expression is showed in red. WT Wilms tumour, CCSK clear cell sarcoma of the kidney, RTK rhabdoid tumour of the kidney, MTSCC mucinous tubular and spindle cell carcinoma, pRCC papillary RCC, cRCC conventional RCC, chRCC chromophobe RCC, RO renal oncocytoma, CDC collecting duct carcinoma. **b**, **c** Expression of the RARRES1 protein at the luminar surface of proximal tubular cells (PT) in foetal and adult kidneys, respectively. No expression was detected in distal tubular cells (DT) or glomerulus (GL). **d** Strong RARRES1 expression at the membrane of conventional RCC showing tubular and trabecular growth pattern. **e** Tumour showing a strong cytoplasmic RARRES1 staining.

The GSEA detected an overexpression of several genes including RARRES1 in conventional RCCs with rapid progression (Fig. 1a). RARRES1 RNA was also highly expressed in the two collecting duct carcinoma, as well as in four papillary RCC with metastatic growth, but no expression was seen in renal oncocytomas and chromophobe RCCs. In this study, we used the data obtained on conventional RCCs.

### Prognostic significance of cellular localisation of RARRES1 protein

To validate the results of gene expression analysis, we used immunohistochemistry on 10 TMA containing 691 conventional RCC. First, we determined the RARRES1 expression in normal foetal and adult kidneys. RARRES1 protein was detected exclusively at the luminal membrane of proximal tubular cells of foetal and adult kidneys (Fig. 1b, c). In tumour biopsies, we detected either a membranous or cytoplasmic RARRES1 expression or no expression. A membranous expression was seen in 454 conventional RCCs displaying tubular or trabecular growth pattern (Fig. 1d). No expression was detected in 106 conventional RCC, whereas 131 tumours showed exclusively cytoplasmic RARRES1 expression (Fig. 1e). As shown in Table 1, the expression of RARRES1 in distinct cellular compartment or lack of its expression was significantly correlated with the size, grade, T classification, necrosis and stage of conventional RCC as well as with postoperative cancer relapse (all *p* < 0.001).

**Fig. 2:**
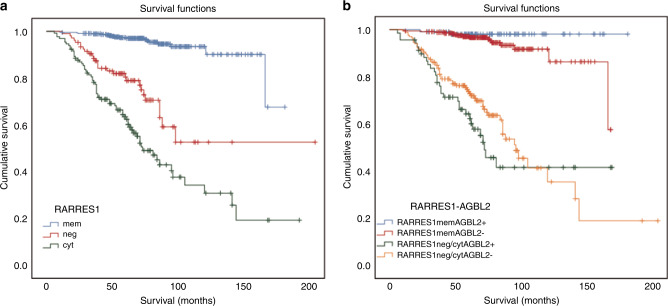
Kaplan–Meier estimates for disease-free survival for patients without metastatic disease at the time of operation. **a** Comparing to the membranous expression, lack of expression or cytoplasmic expression of RARRES1 indicates its prognostic value (*p* < 0.001). **b** Membranous expression of RARRES1 with or without of co-expression of AGBL2 mark a group of tumours with low risk of progression, whereas RARRES1 cytoplasmic/negative and AGBL2-positive/negative expression indicates a high risk of postoperative tumour relapse (*p* < 0.001).

By univariate survival analysis, the T classification, grade, stage, necrosis and size of tumours as well as the RARRES1 expression are significantly associated with tumour progression (all *p* < 0.001). Using multivariate analysis, the cytoplasmic expression or lack of expression of RARRES1 protein was found to be an independent negative prognostic factor indicating 5 times higher risk of cancer relapse (relative risk (RR) = 5.333; 95% confidence interval (CI) = 2.873–9.897; *p* < 0.001 and RR = 4.791; 95% CI = 2.586–8.876; *p* < 0.001, respectively). Subsequent Kaplan–Meier curve analysis confirmed the significant prognostic value of RARRES1 expression at distinct subcellular compartment of conventional RCC without metastasis at the first presentation (Fig. 2a).

### Expression of AGBL2 in conventional RCC

We have applied the carboxypeptidase AGBL2 immunohistochemistry to the series of TMAs that we used for analysis of RARRES1. Cytoplasmic positivity was detected in 180 tumours, whereas 511 ones were negative with the AGBL2 antibody. Each cell of a given tumour designated as positive case displayed a strong immunostaining with the AGBL2 antibody. Evaluation of the AGBL2 expression in relation to the clinical–pathological parameters showed significant correlation only with the tumour grade (*p* < 0.010). Multivariate analysis also revealed that only tumour grade (*p* ≤ 0.002) correlated with AGBL2 positivity. RR for postoperative disease relapse of cases with AGBL2 expression was not significant (RR = 1.096; 95% CI = 0.716–1.677; *p* = 0.672). Subsequent Kaplan–Meier curve analysis confirmed the lack of prognostic significance of AGBL2 expression (*p* < 0.461).

### Co-expression of RARRES1 and AGBL2

The vast majority of AGBL2-positive cases occurred in the group of tumours with RARRES1 membrane positivity (Table 2). Out of 454 conventional RCC with membranous RARRES1 expression, 110 tumours showed AGBL2-positive staining as well. Of the group of 237 RARRES1-negative or cytoplasmic positive tumours, 69 showed AGBL2 positivity, whereas 168 ones were negative for AGBL2. The four combinations of RARRES1 and AGBL2 expression were significantly correlated with tumour size, grade, necrosis, T stadium and stage as well (Pearson Chi-square test, *p* < 0.001). Using multivariate analysis, only tumour necrosis (*p* = 0.017) and combination of RARRES1 cytoplasmic/negative and AGBL2-positive/negative immune reaction showed a significant correlation with survival rates (Table 3). Conventional RCCs with the above combinations of RARRES1 and AGBL2 co-expression have a 11–15 times higher risk for postoperative relapse.

**Table 2:** Correlation between RARRES1/AGBL2 expression and clinicopathological features of RCC analysed by Chi-square test.

	No. of cases (691)	RARRES1/AGBL2 expression				*p* Value
		neg, cyt/− (168)	neg, cyt/+ (69)	mem/− (344)	mem/+ (110)	
Gender						0.002
Male	406	115	48	185	58	
Female	285	53	21	159	52	
Status						<0.001
AWD	579	106	38	327	109	
PTR	112	62	31	17	1	
Size, cm						<0.001
<4	272	31	14	161	66	
4–7	269	73	33	128	35	
>7	150	64	22	55	9	
T						<0.001
pT1	511	89	36	286	100	
pT2	94	30	11	47	6	
pT3	86	49	22	11	4	
Necrosis						
No	608	132	45	323	108	<0.001
Yes	83	36	24	21	2	
Grade						<0.001
G1	456	76	18	279	83	
G2	180	63	32	59	26	
G3	55	29	19	6	1	
Stage						<0.001
I	504	88	35	282	99	
II	167	71	31	57	8	
III	20	9	3	5	3	

*PTR* postoperative tumour relapse.

**Table 3:** Cox regression analysis of clinicopathologic parameters and RARRES1and AGBL2 expression in relation to survival rates.

Parameters	RR	Multivariate analysis (*n* = 691)		
		Relative risk (95% CI)		*p* Value
		Lower	Upper	
Size, cm				
<4				0.555
4–7	0.876	0.447	1.719	0.701
>7	0.670	0.295	1.525	0.340
T				
pT1				0.027
pT2	1.668	0.193	14.384	0.642
pT3	3.272	0.413	25.920	0.262
Grade				
G1				0.188
G2	1.485	0.884	2.495	0.135
G3	1.753	0.947	3.243	0.074
Necrosis	1.705	1.098	2.646	0.017
Stage				
I				0.803
II	2.003	0.245	16.381	0.517
III	1.979	0.260	15.098	0.510
RARRES1^mem^AGBL^pos^				0.000
RARRES1^mem^AGBL^neg^	2.707	0.623	11.767	0.184
RARRES1^neg/cyt^AGBL^pos^	14.658	3.376	63.642	<0.001
RARRES1^neg/cyt^AGBL^neg^	10.985	2.603	46.358	0.001

The Kaplan–Meier analysis of RARRES1 and AGBL2 co-expression in different combinations revealed an excellent prognosis for 110 patients with membranous RARRES1 and cytoplasmic AGBL2 expression (Fig. 2b). Only 1 of the 110 patients developed metastatic tumour during the follow-up. Within the 110 cases, G1, G2 and G3 grade tumours were noticed in 83, 26 and 1 case/s, respectively. One hundred tumours were classified as pT1, 6 as pT2 and 4 as pT3 among the 110 cases with excellent outcome. The only tumour with postoperative relapse was classified as pT3, G3 with necrosis. The 344 RARRES1 membrane-positive and AGBL2-negative tumours displayed identical Kaplan–Meier curve as it was seen for membranous RARRES1 alone (see Fig. 2a). The Kaplan–Meier curves of RARRES1 cytoplasmic/negative and AGBL2-positive/negative cases indicated a strong prognostic value (*p* < 0.001).

## Discussion

The vast majority of 691 conventional RCC without detectable metastasis at the time of operation was classified as pT1a and pT1b tumour indicating per se a low risk of progression. However, 112 tumours developed metastasis during the follow-up. Kaplan–Meier analysis and Cox proportional regression model revealed that cytoplasmic expression of RARRES1 or lack of its expression are significantly associated with tumour progression predicting a five times higher risk for postoperative tumour relapse. Including AGBL2 expression in the evaluation, we found a group of patients with 11–15 times higher risk for postoperative tumour progression. On the other hand, the membranous expression of RARRES1 at the surface of tumour cells marked the large group of patients (454 of 691) with excellent disease outcome. Within this group, 110 tumours displayed a strong cytoplasmic AGBL2 expression in addition to membranous RARRES1 expression. Only one patient from this group presented tumour relapse during the follow-up time. Our observation suggests that RARRES1 may execute distinct biological functions in the same type of tumour depending on its subcellular localisation and co-expression with AGBL2. In contrary to our findings, a recent report described a strong nuclear expression of RARRES1 in clear cell, papillary and chromophobe RCCs.^16^ Zimpfer and colleagues found a correlation between disease outcome and cytoplasmic or nuclear positivity in clear cell RCC. No other studies including ours as well as the examples for RARRES1 immunohistochemistry presented by distinct companies showed nuclear expression.

The RARRES1, or tazarotene-induced gene 1 (TIG1), was identified as highly upregulated gene in skin cultures treated by tazarotene.^21^ Based on the amino acid sequences, RARRES1 is predicted to be a transmembrane protein. Comparing the crystal structure of mouse latexin and to the human latexin homologue RARRES1 also suggested that the basic surface of RARRES1 may interact with membranes.^22^ A cell fractionation study revealed a full-length isoform of approximately 38 kDa RARRES1 as cell membrane bound and another protein of approximately 50 kDa in the nuclear cell debris pool.^6^ RARRES1 have also predicted to localise to other membranes including Golgi apparatus and endoplasmic reticulum (ER). Co-localisation of RARRES1 protein with the ER marker protein disulfide isomerase in LNCaP and PC3 prostate cancer cell lines proved that RARRES1 localises to the ER, whereas RARRES1 was absent from the nucleus.^23,24^

RARRES1 is expressed exclusively at the cell membrane in luminar surface of proximal tubular cells in adult kidney and also in emerging proximal tubules in foetal kidneys. We did not find cytoplasmic RARRES1 expression in any of the normal foetal or adult kidney cells. The vast majority of conventional RCC displayed RARRES1 expression at the membrane of tumour cells, which corresponds to its position in proximal tubular cells. The RARRES1 expression in the cytoplasma may correspond to its presence in the ER. It is possible that only the normal variant of RARRES1 protein can exit from the ER and move to the cell membrane, whereas a highly modified form of 50 KDa RARRES1 or a protein complex containing RARRES1 retained in the ER, e.g. in the cytoplasma of a group of conventional RCCs.

The exact function of RARRES1 in distinct types of cancers including conventional RCC is not yet determined. RARRES1 is a member of a large protein complex including AGBL2, Eg5/KIF11 and EEY bearing protein (EBI), which is involved in the regulation of tubulin tyrosination/detyrosination cycle in tumour cells.^6^ Overexpression of RARRES1 in human embryonal kidney (HEK293) cell line reduced, whereas exogenous expression of AGBL2 increased the level of detyrosinated tubulin.^6^ The α-tubulin detyrosination is associated with tumour progression and poor prognosis.^25^ Using a yeast two-hybrid system, an interaction between RARRES1 and transmembrane protein 192 (TMEM192) has been demonstrated. The RARRES1 expression resulted in cell autophagy via TMEM192, which is expressed in a wide variety of tissues including the kidney.^26,27^ It was also shown that RARRES1 overexpression in PCa cells induces the expression of the autophagy-related genes beclin and ATG3.^28^ RARRES1 has also been associated with diverse biological processes, such as ageing, metabolism and stem cell differentiation.^29^

One interesting finding of our study is the correlation between membranous expression of RARRES1, cytoplasmic expression of AGBL2 and excellent disease outcome. It is possible that co-expression and interacting of membranous RARRES1 with AGBL2 inhibits the α-tubulin detyrosination in this group of conventional RCC resulting in an excellent prognosis. An association between AGBL2 expression and tumour progression has been described in other types of tumours. AGBL2 promotes liver cancer growth via increasing a-tubulin detyrosination, enhancing IRGM-regulated autophagy and inhibiting apoptosis.^30^ Expression of AGBL2 results in α-tubulin detyrosination and cell proliferation of gastric cancer via interaction with latexin.^31^ In our study, a significant association has been seen between AGBL2 expression and tumour grading, which may correspond to the increased α-tubulin detyrosination and proliferative activity of AGBL2-positive tumour cells. A small group of seven patients with AGBL2-positive and RARRES1-negative tumour displayed a shortest survival by Kaplan–Meier curve estimation (data not shown). With all probability, the lack of RARRES1 inhibitor function allows the catalysation of α-tubulin detyrosination by AGBL2 and promotion of tumour progression.

The distinct function of RARRES1 and its interaction with AGBL2 and other genes in conventional RCC as well as in other types of cancers remains to be cleared. In HEK293 cells of mesenchymal origin, RARRES1 is involved in the tyrosination circle of α-tubulin.^6^ However, this function of RARRES1 in MDA-MB-468 breast cancer cell line was excluded.^23^ There is no explanation why the lack of expression of RARRES1 is associated with the progressive growth of colorectal cancer but the increased expression in breast cancer and, as we showed here, its cytoplasmic expression in kidney cancer marks tumour progression.^14,15^ The seemingly controversial functions of RARRES1 might be due to its interaction with different protein complexes and/or its location in different cellular compartments in distinct types of tissue.

In summary, we showed in this study that RARRES1 cytoplasmic/negative and AGBL2-positive/negative staining is a significant risk factor for tumour progression indicating 11–15 times higher risk of cancer relapse, whereas the membranous RARRES1 expression, especially its co-expression with AGBL2, is associated with excellent disease outcome. We suggest that RARRES1 and AGBL2 immunostaining may help to identify a group of patients at a low and high risk of tumour progression and may direct an active surveillance of patients with high risk to detect metastasis as early as possible and stop the follow-up for patients with excellent outcome.

## Data Availability

Data are available upon request.
